# Twelve‐lead and signal‐averaged electrocardiographic parameters among beta‐thalassemia major patients

**DOI:** 10.1002/joa3.12412

**Published:** 2020-07-26

**Authors:** Dimitrios Patsourakos, Konstantinos A. Gatzoulis, Constantina Aggeli, Sophia Delicou, Yannis Dimitroglou, Katerina Xydaki, Konstantinos Toutouzas, Aristeidis Androulakis, Dimitrios Tousoulis

**Affiliations:** ^1^ State Department of Cardiology General Hospital of Athens Ippokrateio Athens Greece; ^2^ First Department of Cardiology General Hospital of Athens Ippokrateio, National and Kapodistrian University of Athens Athens Greece; ^3^ Thalassemia and Sickle Cell Unit General Hospital of Athens Ippokrateio Athens Greece

**Keywords:** beta thalassemia, late potentials, risk stratification, signal‐averaged ECG, sudden cardiac death

## Abstract

**Background:**

The majority of beta thalassemia major (β‐TM) patients suffer from cardiac disease, while a significant proportion of them die suddenly. Twelve‐lead and signal‐averaged electrocardiography (SAECG) are simple, inexpensive, readily available tools for identifying an unfavorable arrhythmiological substrate by detecting the presence of arrhythmias, conduction abnormalities, and late potentials (LPs) in these patients.

**Methods:**

A total of 47 β‐TM patients and 30 healthy controls were submitted to 12‐lead and signal‐averaged electrocardiography. Basic electrocardiographic parameters and prevalence of LPs were recorded. Basic echocardiographic parameters were estimated by transthoracic echocardiography. T2* was calculated by cardiac magnetic resonance imaging wherever available.

**Results:**

β‐TM patients demonstrated a more prolonged PR interval (167.74 msec vs 147.07 msec) (*P* = .043), a higher prevalence of PR prolongation (21.05% vs 0%) (*P* = .013), and a higher prevalence of LPs (18/47, 38.3% vs 2/30, 6.7%) (*P* = .002) compared with controls. The prevalence of atrial fibrillation among b‐TM patients was estimated at 10.64%. Patients had also greater E/e′ ratio (8.35, SD = 2.2 vs 7, SD = 2.07) (*P* = .012) and LAVI (30.7 mL/m^2^, SD = 8.76 vs 24.6 mL/m^2^, SD = 6.57) (*P* = .002) than controls. Regression analysis showed that QTc and LAVI could correctly predict the presence of LPs in the 80.9% of the patients.

**Conclusions:**

β‐TM patients have a higher prevalence of a prolonged PR interval, atrial fibrillation, and LPs. Twelve‐lead and SAECG performance was feasible in all subjects and constitutes a readily available tool for assessing myocardial electrophysiological alterations in this patient group.

## Graphical abstract

A cross‐sectional study considering the value of conventional and signal‐averaged electrocardiography in assessment of the arrhythmiological substrate of β‐thalassemia patients.

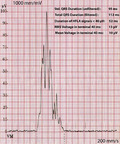

## 1 INTRODUCTION

Thalassemia syndromes are among the most common inherited diseases around the world,^1^ particularly in countries around Mediterranean Sea and Middle East.^2^ Beta thalassemia major (β‐TM) is the most severe type, rendering β‐TM patients transfusion‐dependent lifelong.^3^ The life expectancy of β‐TM patients has increased lately as a consequence of progress in diagnostic and therapeutic protocols,^4^ ranging between 36 and 45 years.^5^ Transfusion dependency leads to iron accumulation in many organs with deleterious consequences.^6^ The heart is, among other organs, affected significantly by excess iron burden.^7^

Cardiac disorders were by far the leading cause of death among β‐TM patients in the past decades, although iron chelation therapy resulted in significant reduction in cardiac related deaths.^8^ Apart from end‐stage heart failure, a significant proportion of β‐TM patients die suddenly.^5^, ^9^

Indeed, according to a recent National Registry in Greece, 10.18% of the β‐TM patients suffered sudden cardiac death.^5^ Atrial fibrillation prevalence among β‐TM patients varies in literature, ranging from 10% to 33.8%.^10^

Signal‐averaged electrocardiography (SAECG) is an attractive method for the arrhythmiological assessment of β‐TM patients, by detecting slow conducting areas in the ventricular myocardium, the so‐called late potentials (LPs). Such abnormal signals have been reported among young β‐TM patients from Lebanon and Italy, ranging from 3 to 31.5%.^11^, ^12^, ^13^ In the same context, previous studies from Greek^14^ and Turkish,^15^ β‐TM patients reported prevalent QRS fragmentation, using 12‐lead ECG.

Taking into account recent progress in diagnostic and therapeutic methods resulting in life prolongation, a new study on β‐TM patients in Greece seems reasonable and promising. The aim of this study is to reveal and describe any electrocardiographic abnormalities in a cohort of Greek β‐TM patients compared with a matched control group, pointing to the presence of an unfavorable arrhythmiological substrate, even at early asymptomatic state.

## 2 METHODS

In the present cross‐sectional study, 47 β‐TM patients who were referred to the Cardiology Unit for Hemoglobinopathies of the Cardiology Department at General Hospital of Athens Ippokrateio and a control group of 30 apparently healthy, age and gender matched, individuals were submitted to standard 12‐lead ECG and SAECG according to a protocol described by ACC expert consensus document.^16^ All patients had been under iron chelation treatment and were examined within a period of 1 week from a scheduled blood transfusion, in order to attenuate the hemodynamic consequences of anemia.

Comorbidities were reported, namely, arterial hypertension, diabetes mellitus, hypothyroidism, and left ventricular dysfunction, based on evidence from clinical examination, laboratory tests, imaging modalities, and concurrent medical treatment. Left ventricular dysfunction was defined as a left ventricular ejection fraction (LVEF) of <50% on recent echocardiographic examination.

Basal rhythm, heart rate, PR interval duration, QRS complex duration and morphology, QTc interval duration, electrical axis of P wave, QRS complex, and T wave were documented from standard 12‐lead ECG, followed by SAECG parameters (fQRS duration, LAS duration, and RMS40 voltage). Presence of at least 2 of the undermentioned criteria set the diagnosis of presence of LPs.^16^

1. fQRS duration ≥114 msec
2. LAS duration ≥38 msec
3. RMS40 voltage ≤20 μV

In case of a prolonged standard QRS duration >120 msec, the modified SAECG criteria were applied.^17^

The acceptable noise level was set at <0.5 μV. QT interval was corrected for heart rate by means of the Bazett equation. QTc > 440 msec in men and >460 msec in women were considered prolonged.

A MAC 5000 resting ECG analysis system (GE MARQUETTE, Fairfield, CT, U.S.A.) was used for all cases.

All participants were examined by transthoracic echocardiography and the following parameters where measured; left ventricular ejection fraction (LVEF), left ventricular end‐diastolic diameter (LVEDD), left ventricular end‐systolic diameter (LVESD), ratio between early mitral inflow velocity and mitral annular early diastolic velocity (E/e'), left atrial volume index (LAVI), and pulmonary artery systolic pressure (PASP). A Philips EPIQ 7C Cardiology Ultrasound System (Philips Medical Systems, Andover, MA, USA) was used.

Cardiac magnetic resonance (CMR) T2* values were available for 38 of 47 patients, while the remaining nine patients could not be examined by CMR as a result of either patient unwillingness or contraindications. Serum ferritin levels were documented according to recent (<3 months) laboratory testing.

Informed consent was obtained from every individual participated in the present study, which conforms to the ethical guidelines of the 1975 Declaration of Helsinki and complies with the principles of General Data Protection Regulation (GDPR). The human research and bioethics committee of the affiliated health institution have granted permission for the current study.

Continuous variables were presented using measures of central tendency and variability, including the mean and standard deviation (SD) or median and interquartile range (IQR, 25th‐75th) as appropriate, while ordinal or nominal variables were presented as numbers or percentages. Student’s *t*‐test was used for parametric and Fisher’s exact test for categorical variables. Logistic regression models were built to determine, whether clinical, electrocardiographic, and echocardiographic parameters could be used as predictors of the presence of LPs. Age, gender, and all parameters with an initial probability of type b error (*P*‐value) less than .15 were included in the initial model. Consequently, backward conditional elimination of the variables was used to assess for those with higher significance when predicting the presence or not of LPs. Variables with a *P*‐value higher than .10 were excluded for the model. A *P*‐value of ≤.05 was considered statistically significant. SPSS 23 statistics software (IBM—Armonk) was used for statistical analysis.

## 3 RESULTS

### 3.1 Demographics and comorbidities

Basic demographic characteristics and comorbidities are demonstrated in Table 1. Study patients and controls were age and gender matched, while none of the patients had arterial hypertension. Nine patients had diabetes mellitus (19.1%), 14 patients had hypothyroidism (29.8%), and four patients had left ventricular dysfunction (8.5%). Ferritin median in patient group was 880 μg/L [340‐2300]. All patients with the aforementioned comorbidities were receiving appropriate treatment with no sign of decompensated organ dysfunction.

**TABLE 1:** Basic demographic characteristics, comorbidities, and echocardiographic parameters among patients and controls

Parameter	Patients (n = 47)	Controls (n = 30)	*P* for difference
Age (y)	40.91 (SD = 7.17)	41 (SD = 14.1)	.972
Male gender (n, %)	21 (51.1%)	14 (46.6%)	.707
Serum ferritin (μg/L)	880 [340‐2300]		
Cardiac T2* (msec)	32.7 (SD = 9.24)		
Arterial hypertension	0	0	
Diabetes mellitus	9 (19.1%)	0	.011
Hypothyroidism	14 (29.8%)	0	.001
Left ventricular dysfunction	4 (8.5%)	0	.101
LVEF (%)	59.7 (SD = 4.6)	61 (SD = 4.8)	.232
LVEDD (mm)	49.1 (SD = 4.26)	47.83 (SD = 3.7)	.190
LVESD (mm)	32.1 (3.96)	32.1 (2.96)	.966
E/e′	8.35 (SD = 2.2)	7 (SD = 2.07)	.012
LAVI (mL/m^2^)	30.7 (SD = 8.76)	24.6 (SD = 6.57)	.002
PASP (mm Hg)	28.32 (4.06)	27.67 (5.37%)	.547

Note

Data are presented as means (SD), median [IQR], or N (%).

Abbreviations: E/e′, ratio between early mitral inflow velocity and mitral annular early diastolic velocity; LVEDD, left ventricular end‐diastolic diameter; LVEF, left ventricular ejection fraction; LVESD, left ventricular end‐systolic diameter; LAVI, left atrial volume index; PASP, pulmonary artery systolic pressure.

### 3.2 Electrocardiography

Regarding basic electrocardiographic parameters (Table 2), all patients and controls were in sinus rhythm. Five patients reported documented episodes of paroxysmal atrial fibrillation (prevalence = 10.64%). Heart rate did not differ significantly between the two groups, while the measured PR interval of β‐TM patients was greater than controls (167.74 msec, SD = 52 vs 147.07 msec, SD = 22.25) (*P* = .043). Six men and two women (eight patients in total, 17.02%) had PR interval prolongation. No one in control group had a prolonged PR interval (*P* = .017). Neither QRS complex duration nor QTc interval duration differed significantly between patients and controls. According to the aforementioned criteria, one man and one woman in the patient group had a prolonged QTc interval (4.25%) while no one in the control group was found with QTc prolongation (*P* = .252). As far as electrical axis is concerned, no significant axis deviation of P wave, QRS complex, or T wave was noticed.

**TABLE 2:** Basic and signal‐averaged electrocardiographic parameters among patients and controls

Parameter	Patients (n = 47)	Controls (n = 30)	*P* for difference
Heart rate (bpm)	77.32 (SD = 14.49)	73.80 (SD = 15.5)	.296
PR interval (msec)	167.74 (SD = 52)	147.07 (SD = 22.25)	.043
PR interval prolongation (dichotomous) n, %	8 (17.02%)	0 (0%)	.017
QTc duration (msec)	421.9 (SD = 21.36)	423.7 (SD = 21.45)	.716
QTc prolongation (dichotomous) n, %	2 (4.25%)	0 (0%)	.252
QRS duration (msec)	93.8 (SD = 12.86)	89.93 (SD = 8.53)	.152
P‐wave axis (°)	54.85 (SD = 14.39)	52.53 (SD = 17.78)	.532
QRS complex axis (°)	51.3 (SD = 29.66)	51.6 (SD = 37.22)	.963
T‐wave axis (°)	45.28 (SD = 17.85)	53.57 (SD = 15.73)	.119
fQRS duration (ms)	114.94 (SD = 13.23)	108.93 (SD = 9.7)	.035
LAS duration (ms)	37.94 (SD = 12.82)	31.33 (SD = 6.02)	.01
RMS (μV)	28.85 (SD = 16.45)	37.6 (SD = 13.96)	.018
LPs presence (n, %)	18 (38.3%)	2 (6.7%)	.002

Note

Data are presented as means (SD), median [IQR], or N (%).

Abbreviations: fQRS, filtered QRS; LAS, low‐amplitude signals; LPs, late potentials; RMS, Root mean square QRS size at final 40 msec.

### 3.3 SAECG

SAECG parameters of both groups are presented in Table 2. The average noise level was 0.33 μV (SD = 0.046 μV). The prevalence of LPs was significantly higher among β‐TM patients, compared with controls (18/47, 38.3% vs 2/30, 6.7%) (*P* = .002). Every single SAECG parameter significantly differed among patients compared with controls. In particular, filtered QRS duration was more prolonged among patients than controls (114.94 msec, SD = 13.23 vs 108.93 msec, SD = 9.69) (*P* = .035), low‐amplitude signals duration was more prolonged among patients than controls (37.94 msec, SD = 12.82 vs 31.33 msec, SD = 6.02) (*P* = .01), and root mean square QRS voltage at final 40 msec was lower among patients than controls (28.85 μV, SD = 16.45 μV vs 37.6 μV, SD = 13.96 μV) (*P* = .018).

### 3.4 Echocardiography

Regarding basic echocardiographic parameters (Table 1) LVEF, LVEDD, LVESD, and PASP did not differ significantly between patients and controls. On the contrary, the ratio E/e′ was greater among patients (8.35, SD = 2.2) compared with controls (7, SD = 2.07) (*P* = .012). Similarly, the left atrial volume index (LAVI) in patient group was greater than controls (30.7 mL/m^2^, SD = 8.76 vs 24.6 mL/m^2^, SD = 6.57) (*P* = .002).

### 3.5 CMR

CMR data were available for 38 of 47 patients. The mean value of T2* was 32.7 msec (SD = 9.24), depicting the fact that the majority of the patients had normal myocardial iron burden, as assessed by T2* value. Regarding the eight patients with a prolonged PR interval, CMR data were available for half of them, having normal values of T2* (36.2, 34.5, 35.0, and 36.1 msec, respectively).

### 3.6 Characteristics of LP‐positive and LP‐negative patients

Table 3 summarizes the demographic characteristics, comorbidities, electrocardiographic, echocardiographic, and CMR findings among patients according to the presence or absence of LPs. Age did not differ significantly between LPs‐positive and LPs‐negative patients. Neither hypothyroidism nor diabetes mellitus was more prevalent among LP‐positive patients.

**TABLE 3:** Demographic characteristics, electrocardiographic, echocardiographic, and CMR parameters in patient group based on the presence of LP

Parameter	Presence of LP (n = 18)	Absence of LP (n = 29)	*P* for difference
Male gender (n, %)	12 (66.7%)	12 (41.4%)	.092
Age (y)	42.72 (SD = 6.6)	39.79 (SD = 7.4)	.177
Diabetes mellitus	3 (16.67%)	6 (20.69%)	.733
Hypothyroidism	8 (44.4%)	6 (20.7%)	.083
Left ventricular dysfunction	2 (11.1%)	2 (6.9%)	.615
Heart rate (bpm)	73.94 (SD = 12.8)	79.4 (SD = 13.68)	.179
PR interval duration (msec)	182.56 (SD = 73.5)	158.55 (SD = 30.5)	.125
QRS duration (msec)	101.22 (SD = 12.87)	89.17 (SD = 10.66)	.001
QTc duration (msec)	429.9 (SD = 24.8)	416.9 (SD = 17.5)	.041
P wave axis (°)	51.5 (SD = 17.1)	56.9 (SD = 12.3)	.212
QRS axis (°)	47.3 (SD = 38.8)	53.72 (SD = 22.7)	.475
T‐wave axis (°)	42.6 (SD = 22.2)	50.2 (SD = 14.2)	.16
fQRS duration (ms)	123.1 (SD = 12.25)	109.9 (SD = 11.3)	<.001
LAS duration (ms)	50.22 (SD = 11.8)	30.31 (SD = 5.3)	<.001
RMS (μV)	13.61 (SD = 4.4)	38.3 (SD = 13.8)	<.001
LVEF (%)	58.33 (SD = 4.54)	61 (SD = 4.6)	.093
LVEDD (mm)	50.94 (SD = 4.77)	47.93 (3.53)	.017
LVESD (mm)	33.28 (SD = 4.38)	31.31 (SD = 3.55)	.098
E/e′	8.98 (SD = 2.02)	7.96 (SD = 2.25)	.123
LAVI (mL/m^2^)	35.83 (SD = 10.12)	27.5 (SD = 6.03)	.001
PASP (mm Hg)	30 (SD = 4.85)	27.28 (SD = 3.14)	.024
Ferritin (μg/L)	571 [269‐1900]	977 [370‐2400]	.242
Cardiac T2* (msec)	32.11 (SD = 10.63)	33.16 (SD = 8.33)	.736

Note

Data are presented as means (SD), median [IQR], or N (%).

Abbreviations: E/e′, ratio between early mitral inflow velocity and mitral annular early diastolic velocity; fQRS, filtered QRS; LAS, low‐amplitude signals; LAVI, left atrial volume index; LVEDD, left ventricular end‐diastolic diameter; LVEF, left ventricular ejection fraction; LVESD, left ventricular end‐systolic diameter; PASP, pulmonary artery systolic pressure; RMS, Root mean square QRS size at final 40 msec.

Heart rate, electrical axis, and PR interval did not differ between the subgroups, while QTc duration was more prolonged among LPs‐positive patients (429.9 msec, SD = 24.8 vs 416.9 msec, SD = 17.5) (*P* = .041). Regarding SAECG parameters, filtered QRS duration was found more prolonged among LPs‐positive patients compared with LPs‐negative patients (123.1 msec, SD = 12.25 vs 109.9 msec, SD = 11.3) (*P* < .001) as well as low‐amplitude signals duration (50.22 msec, SD = 11.8 vs 30.31 msec, SD = 5.3) (*P* < .001). In addition, root mean square QRS voltage at final 40 msec was found lower among LPs‐positive patients compared with LPs‐negative patients (13.61 msec, SD = 4.4 vs 38.3 msec, SD = 13.8) (*P* < .001).

Νeither LVEF nor LVESD differed significantly between the subgroups. On the contrary, LVEDD was significantly greater in patients with LPs compared with patients without LPs (50.94 mm, SD = 4.77 vs 47.93 mm, SD = 3.53) (*P* = .017). The E/e′ ratio did not differ significantly between the subgroups, as opposed to LAVI that was found significantly increased in patients with LPs (35.83 mL/m^2^, SD = 10.12 vs 27.5 mL/m^2^, SD = 6.03) (*P* = .001). Similarly, PASP was greater among patients with LPs (30 mm Hg, SD = 4.85 vs 27.28 mm Hg, SD = 3.14) (*P* = .024).

Keeping in mind that CMR data were lacking for nine patients in our study, statistical analysis did not reveal significant difference between patients with LPs and patients without. Similarly, ferritin levels did not differ among the subgroups.

Age, gender, history of hypothyroidism, QTc, LVEDD, LVEF, LAVI, E/e′, and PASP were included in the initial multiple logistic regression model. The model could correctly classify 85.1% of the patients with an R^2^ of 0.562. QTc was the only significant predictor for the presence of LPs. In the final model, QTc and LAVI were included and presence of LPs could be correctly predicted in the 80.9% of the patients (Table 4).

**TABLE 4:** Multivariate logistic regression analysis of the clinical, electrocardiographic, and echocardiographic predictors of the presence of LPs

Variables	Initial model			Final model (step 7)		
	OR	95% CI	*P*‐value	OR	95% CI	*P*‐value
Male gender	2.790	0.283‐27.551	.380			
Age	0.991	0.875‐1.122	.882			
Hypothyroidism	2.723	0.381‐19.482	.318			
QTc	1.054	1.005‐1.105	.031	1.046	1.010‐1.084	.004
LVEF	0.939	0.733‐1.203	.620			
LVEDD	1.134	0.849‐1.515	.395			
E/e′	1.306	0.876‐1.949	.191			
LAVI	1.142	0.933‐1.398	.197	1.202	1.059‐1.364	.004
PASP	1.024	0.789‐1.329	.869			

Abbreviations: E/e′, ratio between early mitral inflow velocity and mitral annular early diastolic velocity; LAVI, left atrial volume index; LVEDD, left ventricular end‐diastolic diameter; LVEF, left ventricular ejection fraction; PASP, pulmonary artery systolic pressure.

### 3.7 Follow up

**FIGURE 1:**
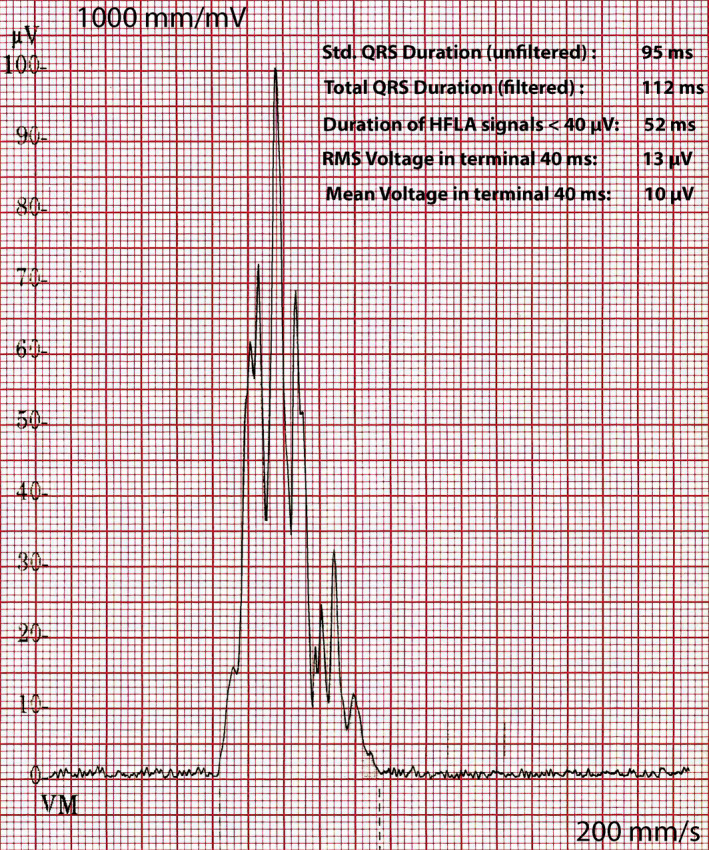
Signal‐averaged electrocardiogram of a 42‐y‐old, female, β‐TM patient. Note the low‐voltage signals at the terminal portion of the filtered QRS complex. All criteria are fulfilled (3/3) for the presence of late potentials (see main text)

**FIGURE 2:**
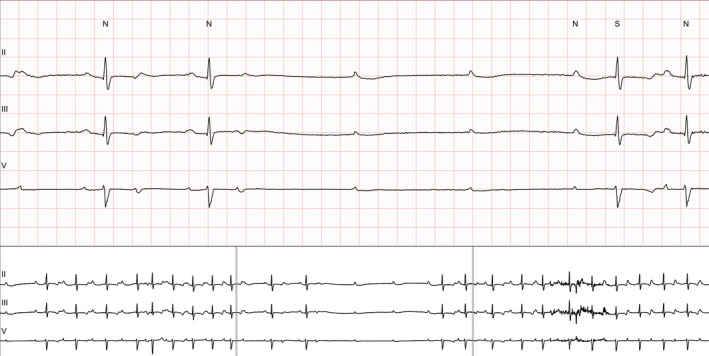
Continuous Holter electrocardiography recording revealing an episode of high‐degree atrioventricular block with an asystole period of 4.2 s during daytime

**FIGURE 3:**
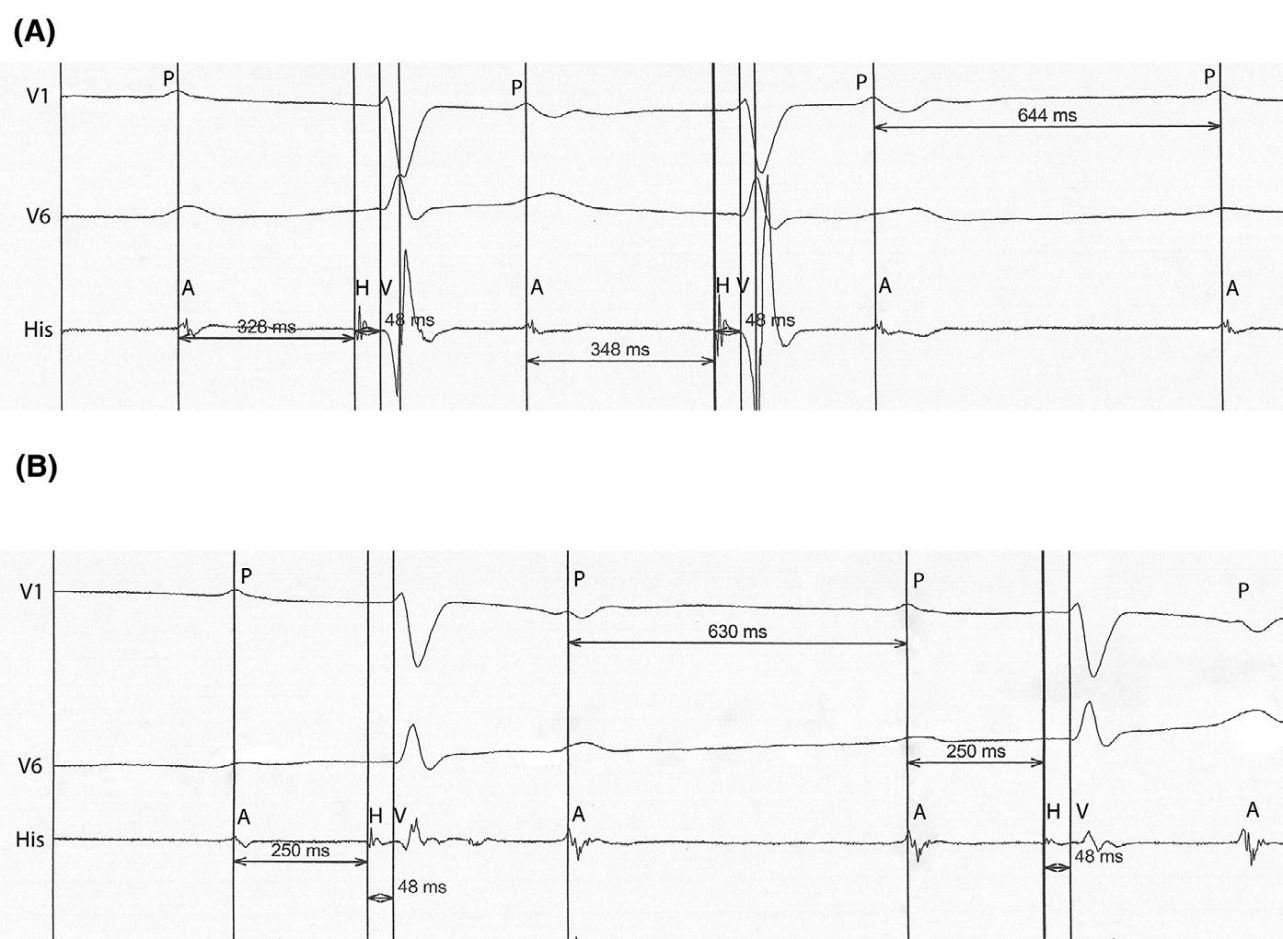
Recordings from electrophysiological study. Tracing A shows intranodal suprahisian Wenckebach at 644 msec cycle length. Tracing B shows intranodal suprahisian 2:1 atrioventricular block at 630 msec cycle length. Note that the HV interval remains within normal limits (48 msec). HIS, His bundle catheter

**FIGURE 4:**
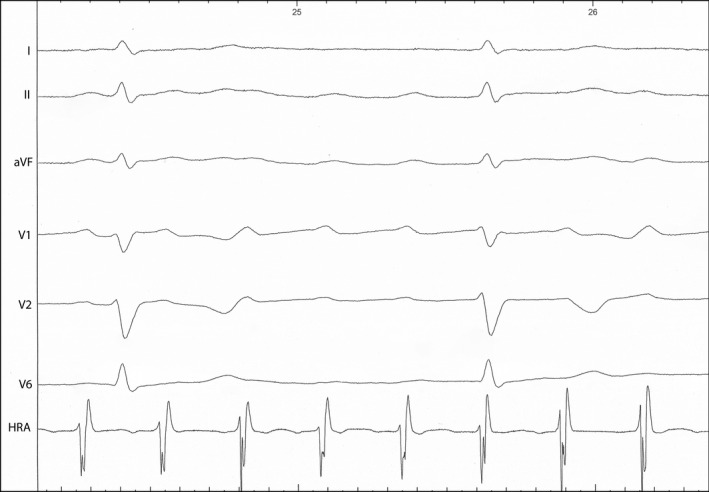
Recording from electrophysiological study showing the induction of atrial flutter with high‐degree atrioventricular block during programmed atrial stimulation. HRA, High right atrial catheter

During the first year of follow up, one patient developed symptoms. A 42‐year‐old female, with a history of β‐TM, diabetes mellitus type 2, and two episodes of paroxysmal atrial fibrillation (the most recent 6 years ago), reported new onset of dizziness, 2 months after the first evaluation for the current study. The baseline ECG had shown a prolonged PR interval, incomplete RBBB, and nonspecific T‐wave abnormalities in precordial leads. The echocardiographic examination revealed normal findings, apart from a moderately elevated LAVI (38 mL/m^2^) and PASP (40 mm Hg). She was not receiving medication with negative chronotropic, dromotropic, or inotropic effect. The SAECG demonstrated 3/3 criteria for the presence of LPs (Figure 1). The patient had no evidence of myocardial iron overload according to recent CMR (T2* = 36.1 msec). Continuous Holter electrocardiography revealed an episode of high‐degree atrioventricular (AV) block with an asystole period of 4.2 sec, recorded during daytime (Figure 2). An electrophysiological study (EPS) was conducted, which revealed the following (Figure 3): AH interval = 250 msec, HV interval = 48 msec, as well as spontaneous appearance of second‐degree AV block caused by suprahisian intranodal block. Atrial flutter with high‐degree AV block was induced during programmed atrial stimulation (Figure 4), while ventricular tachycardia was not induced during programmed ventricular stimulation. A dual‐chamber pacemaker was successfully implanted.

## 4 DISCUSSION

The main finding in our study is that β‐TM patients have a higher prevalence of a prolonged PR interval (17.02%), atrial fibrillation (10.64%), and LPs (38.3%). These findings are in agreement with previous reports.^9^, ^10^, ^11^, ^12^, ^13^, ^18^ However, it is the first study that quantifies the prevalence of PR interval prolongation and LPs in a β‐TM population of advanced age (mean value 40.91 years) compared with previous studies.^11^, ^12^, ^13^ Additionally, our study is unique in terms of combining data from patient comorbidities, electrocardiography, echocardiography, and CMR.

Studies concerning the prevalence of conduction abnormalities among β‐TM populations are lacking. Conduction disturbances are thought to occur at a latter stage of iron overload cardiomyopathy.^19^ The recent progress in medical treatment of these patients heralds prolongation of life expectancy and consequently higher prevalence of conduction abnormalities than has been previously recognized. The current study demonstrated that β‐TM patients exhibit PR interval prolongation, indirectly implying the presence of atrioventricular conduction impairment. Although first‐degree atrioventricular block per se does not consist a direct indication for further examination, β‐TM patients with this kind of disorder should be thoroughly questioned about related neurological symptoms and monitored by repeated 12‐lead ECGs or even ambulatory continuous ECG recording.^20^

An interesting observation is the fact that patients with PR prolongation were found having a normal level of cardiac iron concentration, as this was expressed by CMR T2* value. Despite the fact that CMR data were available for only half of these patients, it seems that some degree of conduction impairment remains despite achieving an acceptable level of cardiac iron concentration following appropriate iron chelation therapy.^21^ The clinical value of this finding is descriptively depicted on the aforementioned example of a previously asymptomatic patient with a prolonged PR interval that eventually became symptomatic during the follow‐up period, while receiving adequate chelation therapy, as was evident by CMR T2* value.

The estimated prevalence of atrial fibrillation in this population (5/47, 10.64%) complies with similar observations from former studies. However, the true prevalence may be even higher if long‐term ambulatory ECG recordings were available among our β‐TM patient population.

The echocardiographic evaluation of both groups revealed that β‐TM patients had impaired diastolic indices, as expressed by increased E/e′ ratio and LAVI. This is not a new finding, but is consistent with current knowledge that diastolic dysfunction is the earliest myocardial complication observed in β‐TM patients.^5^ In a previous study from our Cardiology Unit for Hemoglobinopathies, we had shown, using 3D echocardiographic techniques, that the volume of the left atrium was increased in asymptomatic β‐TM patients.^22^

The higher prevalence of LPs among β‐TM patients compared with controls may be attributed to the deleterious effects of myocardial iron deposition. Our reports are consistent with the study from Italy,^13^ which included fewer (n = 19) and younger (18.4 ± 8.3 years) patients reporting a prevalence of LP at 31.5%. However, that study did not reveal any relation between LPs and other electrocardiographic or echocardiographic parameters. On the contrary, our study demonstrated a correlation between LPs and LVEDD, LAVI and QTc interval duration.

Additionally, our study shows that while the presence of LPs is independent of certain common comorbidities (diabetes mellitus, hypothyroidism), it is related not only to the size of the left ventricle but also to the size of the left atrium, the pulmonary artery systolic pressure and the QTc interval duration.

A positive correlation between LPs and left ventricle dimensions was also observed in a Lebanese study.^12^ However, a much lower prevalence of LPs was reported in this study. The higher LPs prevalence in our study may be attributed to the advanced age of our β‐TM population (40.91 ± 7.17 years).

Regarding sudden cardiac death risk estimation, conventional prognostic tools may be used, namely, left ventricular ejection fraction (LVEF), the most commonly used tool for risk stratification in ischemic, and dilated cardiomyopathy. However, in β‐TM patients, systolic dysfunction is not included among the earliest manifestations of cardiac iron deposition.^19^, ^23^ Our β‐TM patients with LPs had a slightly lower LVEF and more dilated left ventricle, suggesting the presence of a vulnerable for ventricular arrhythmias myocardial substrate. Indeed, it has been shown that an inhomogeneous pattern of myocardial iron deposition in such patients creates areas of slow conduction serving as substrate for re‐entry ventricular arrhythmias.^19^, ^24^, ^25^

The present study suggests a correlation between a prolonged QTc duration and an increased left atrial volume with the presence of LPs in β‐TM population. Future long‐term observational studies will establish a more definite correlation of LPs with age, echocardiographic indices, cardiac magnetic resonance parameters, as well as with the occurrence of potentially malignant or even malignant ventricular arrhythmias.

The cross‐sectional nature of the current study is its major limitation. Long‐term observational studies are under way incorporating sophisticated imaging modalities such as novel echocardiographic techniques (speckle tracking imaging) and cardiac magnetic resonance imaging. Data from CMR were available in 38 of 47 patients, preventing us from drawing more reliable conclusions regarding the correlation between T2* and other electrocardiographic and echocardiographic indices. Another limitation is the relatively small number of patients. Thalassemia intermedia patients, being an heterogeneous group regarding transfusion dependency, were excluded from the current study, thus favoring the extraction of grossly abnormal results.

## 5 CONCLUSIONS

The current study attempts to bring electrical conduction abnormalities among β‐TM patients to the foreground, an area that has been neglected until now, since the majority of studies focused on systolic or diastolic impairment of the heart function. The demonstration of higher prevalence of PR interval prolongation and LPs among β‐TM patients experiencing significant life prolongation nowadays calls for closer patient surveillance and increased physician alertness. It is likely that the timely application of appropriate treatment may have significant impact on survival and quality of life in this unique cardiomyopathy patient population as well.

## CONFLICT OF INTEREST

Authors declare no conflict of interests for this article.
